# Case of Extra-Uterine Fœtation

**Published:** 1831

**Authors:** 


### XXVI.

CASE OF EXTRA-UTERINE FŒTATION. By *M. F. Wagstaff*, Esq.; with a Plate.

"ON the 28th of April, 1829, I was desired to visit Mrs. RONDEAU, a married lady, about twenty-five years of age. I had delivered her of twins in her first accouchement, and of a single child in her second, which child, at this period, had not been weaned. No irregular circumstances occurred in either of those labours.

I found her reclined on a sofa, on the right side, her countenance very pallid, pulse greatly accelerated and small, much anxiety about the præcordia, lancinating pains in the abdomen, constant tenesmus both of rectum and bladder, with frequent vomiting.

Her mother gave the following statement of the previous occurrences :—

'After having eat some cold lamb and potatoes, and drank a glass of porter, we walked together into Blackfriars' road, (about half a mile), when Mrs. RONDEAU *suddenly* complained of *acute* pain in the body, and requested a coach to be procured, in which she was conveyed home, where I gave my daughter some warm brandy and water immediately, supposing the pain to have arisen from indigestion, especially as it was attended with vomiting and relaxation of the bowels, and applied warm fomentation : this took place at two o'clock ; at four, (as I found the pain increase,) I sent for you.'

The symptoms, as they appeared on my arrival, I have just described : they were very analogous to those of Cholera Morbus, but, as no inflammatory symptoms supervened, I prescribed anodyne and carminative medicines.

The pain continued during the whole evening; the abdomen enlarged, the pulse diminished, and, a little before twelve the same night, she expired.

The rapid progress and fatal termination of this case naturally excited a desire to be better acquainted with the *cause* of dissolution, which was not clearly shown by the SYMPTOMS ; I, therefore, requested permission to inspect the body, which was readily granted by the relatives.

On the subsequent afternoon I went, accompanied by Mr. NORDBLAD, Curator of St. Thomas's Museum, and my Son, to make an examination, of which the following is the statement :—

### SECTIO CADAVERIS.

On the appearance of the body, fourteen hours after death, the surface exhibited a completely blanched state, with fulness and tension of the abdomen.

On cutting through the abdominal muscles, the peritoneal sack was found to be distended with *blood*, (amounting, by weight, to four pounds fourteen ounces,) partially coagulated.

While this was being removed, cautiously, a fœtus was discovered in it of about seven or eight weeks' growth.

No laceration appearing in the uterus, attention was naturally directed to the appendages of that viscus, when the part where the fœtus had escaped was manifested by the ragged placenta, which hung from its situation in the left fallopian tube.

This left no doubt as to the nature of the case. The parts were then removed, and on a careful examination, it was ascertained that the fœtus had been contained in a sac formed by the left fallopian tube.

This sack was equal in size to a pigeon's egg, and occupied two-thirds of the length of the tube, nearest to the uterus.

The remaining portion of the tube was of its natural size, and its fimbriated margin free from obstruction.

A probe passed along its canal, stopped abruptly at the commencement of the dilatation, where the placenta protrudes; the distention of its sac increased to its centre, at which part it had given way, allowing the exit of the fœtus.

The rupture is as large as the point of a finger, and through it a detached portion of the placenta hung into the abdominal cavity.

The body of the uterus had enlarged to a size equal to that of the same viscus at a similar period of gestation; its walls were thickened and its cavity lined by recent deposit, evidently forming the decidua.*

A careful examination could not detect any rent in the peritoneal surface, nor was there the slightest mark of visceral disease.

* "This is a peculiar circumstance, worthy of investigation. How does it happen that the decidua should be *forming in* the uterus when the fallopian tube was imperforate *beyond* the fœtus. No communication whatever between it and the uterus. The uterus had taken on its action of pregnancy although empty, and a deposit to form, or forming a membrane.

Is this by the excitement of aura or by sympathy and consent of parts?"

REMARKS.

Extra-Uterine Fœtations are probably more frequent than we have been accustomed to imagine: little was known about them previous to the last century.

Mr. Turnbull, a surgeon of eminence, published a case of Extra-Uterine Fœtation, of the ventral kind, where pregnancy had existed fifteen months, and the child was *(post mortem)* removed by him from the abdomen. In this publication he refers as far back as 1683, to a case published in the Philosophical Transactions of that date, and gives a list of references to various authors, down to the days of Drs. Smellie, Lowther, and Garthshore, and so on to his own case, published in 1791.

He remarks, 'that, although it is generally understood that the uterus is essentially necessary for the purposes of conception, yet these different fœtations incline us to believe that it is not *absolutely so*, and that the principal advantages which that organ possesses over other living parts are derived from its situation and dilatable powers, and from its being possessed of *muscular* structure with an *external opening*. The former being admirably calculated for the purposes of growth and evolution, without any interference with the vital parts; and the latter for the prevention of hemorrhages and the expulsion of the fœtus.'

The case I have just related seems calculated to corroborate this hypothesis. Does it not prove that the uterus is only a receptacle? though evidently the best suited for the purpose (being the natural one); yet, in this particular instance, the fœtus was nourished seven or eight weeks in its unnatural habitation—the fallopian tube, which, being impervious at the uterine extremity, could not permit the passage of the fœtus into the uterus.

A question here naturally arises, How was the fœtus nourished? It continued to enlarge until the texture of the fallopian tube gave way to over-distention.

It is evident that its nourishment was obtained from the vessels of the placenta, which received it from their numerous ramifications on the internal surface of the fallopian tube. Suppose, in the expulsion of the fœtus through the rupture in the fallopian tube, the funis umbilicalis had *not* been separated, but remained perfect, would it not have continued to supply the fœtus from the same source it had done before, namely, the internal surface of the fallopian tube; and might not its ramifications have been extended over neighbouring parts, and thereby produced a supply, augmenting with the enlargement and demand of the fœtus?

The generally-received theory is, that the ovum is impregnated in the ovarium and becomes detached in consequence; the fimbriæ of the fallopian tube, having seized the ovarium, conveys the ovum, at the moment of its detachment, into the uterus, for nourishment; but, if they should miss their grasp,—or, having obtained it, should loose their hold, it must fall into the abdominal cavity, and thereby constitute a ventral case.

Adhesion readily takes place, from which ramifications are soon found to extend, and obtain a supply for the nourishment and growth of the fœtus.

In *this case*, however, it would appear that the impregnated ovum had been conveyed *(by the fimbrial grasp)* into the fallopian tube, but, meeting with obstruction there, it remained until its enlargement caused the tube to be ruptured: yet it had formed ramifications *within* the tube, from which it received its nourishment.

To the rupture of the funis, &c.—partial separation of the maternal portion of the placenta—are to be attributed the death of the patient, for nearly five pounds of blood were found loose in the cavity of the abdomen.

When we consider the smallness of the funis at that early period of pregnancy, we cannot reconcile that a sufficient quantity should have been so rapidly extravasated from the funis alone as to terminate fatally.

We have to regret that, had we even been acquainted with the symptoms, we were not likely to remedy the evil. Speculative theory might induce a bold surgeon to divide or tie the fallopian tube, but with what result is perfectly conjectural.

The case I have just described must for ever have remained in obscurity, except for a *post mortem* examination. I, therefore, congratulate the Profession on the advantages which the present age affords over former times to the pursuit of pathological inquiries.

The occurrences of peculiar phenomena,—deviations from the laws of the animal economy,—alteration in the structure and functions of any of the organs of the human body by disease,—are at all times peculiarly interesting to the contemplative Physiologist and Pathologist."

---

